# Supplementary material for: Electrostatic control of the proximity effect in the bulk of semiconductor-superconductor hybrids
Source: Nat Commun. 2023 Jun 7;14:3325. doi: 10.1038/s41467-023-39044-w (PMC10247816; doi:10.1038/s41467-023-39044-w)
Supplement: Supplementary file 1 — Supplementary information [file 41467_2023_39044_MOESM1_ESM.pdf]

Supplementary information: Electrostatic control of the proximity effect in the bulk of semiconductor-superconductor hybrids

Nick van Loo[‡,1] Grzegorz P. Mazur[‡,1,*] Tom Dvir,[1] Guanzhong Wang,[1] Robin C. Dekker,[1] Ji-Yin Wang,[1] Mathilde Lemang,[1] Cristina Sfiligoj,[1] Alberto Bordin,[1] David van Driel,[1] Ghada Badawy,[2] Sasa Gazibegovic,[2] Erik P.A.M. Bakkers,[2] and Leo P. Kouwenhoven[1,†]

[1]*QuTech and Kavli Institute of Nanoscience Delft University of Technology, 2600 GA Delft, The Netherlands*

[2]*Applied Physics department, 5600 MB Eindhoven, The Netherlands*

(Dated: May 8, 2023)

I. ANALYSIS DETAILS

A. Determination of the induced gap and gap in the superconducting shell

In Fig. 1 we show how we extract Δ_i and Δ_SC from the nonlocal conductance. Fig. 1a shows the nonlocal conductance g_LR from the $8\,\mu$m device, which is also shown in the main text Fig. 2. We show examples of the extraction algorithm for two linecuts, presented in the middle column (yellow) and right column (orange) for a large gap and a closed gap, respectively. In panels Fig. 1c and g, the two linecuts are shown. We first split the signal into two separate traces for positive (red) and negative (blue) biases and take its absolute value, as shown in Fig. 1d and h. Next, the two traces are normalized by their peak value. We then look at the noise level of each trace for biases larger than Δ_SC, as shown in dark blue and dark red in Fig. 1e and i. The maximum of the noise level is then used to set a threshold value, shown as the horizontal lines in dark blue and dark red in Fig. 1e and i. We then apply this threshold value to the Savitzky-Golay filtered version of the data as shown in Fig. 1f and j, which estimates Δ_i individually for positive and negative bias as shown in light green and pink vertical lines. Similarly, the threshold is used to estimate Δ_SC as shown in dark green and purple vertical lines in Fig. 1f and j. Finally, we show the filtered and renormalized data in Fig. 1b, together with the four estimated energy values Δ_i and Δ_SC at positive and negative biases. We see that the algorithm estimates them very well from the nonlocal signal, but sometimes deviates. This is usually the result of a linecut with a weak signal, which cannot be avoided during these measurements. We always check by eye if the obtained energy values match the nonlocal signals well. We occasionally increase the threshold for the estimation of the gaps by a factor of ~ 1.2 - 3 in order to avoid false triggers on noise, which is typically needed for longer nanowires where the magnitude of the nonlocal signal is small. Similarly, we apply a maximum to the threshold of ~ 0.4 - 0.8 to prevent the algorithm from failing to find a value.

We apply the above algorithm to both nonlocal signals g_RL and g_LR of a given measurement. From this we obtain four estimates of Δ_i: one at positive and one at negative bias, for both g_RL and g_LR. This is shown in Fig. 2a, which displays both nonlocal conductances from device B ($8\,\mu$m) together with the extracted Δ_i estimates. In the top panel of Fig. 2b, the four Δ_i traces are displayed as a function of super gate voltage V_SG. We take the mean value of these traces as Δ_i, which is shown in dark blue in the bottom panel of Fig. 2b. In addition, we take the standard deviation of the four traces and plot it as the shaded blue area. A small standard deviation means the four estimates agree well to one another, which increases our confidence that the algorithm extracts the correct values. However in some cases, the nonlocal signal is strongly asymmetric in terms of signal strength which can hinder the correct estimation of one of the four traces. We always check by eye if this happens, in which case we adjust the procedure to first omit the strongly deviating trace out of the two positive-bias and negative-bias traces for a single nonlocal matrix element. Subsequently, one trace for g_RL and one trace for g_LR remain of which the mean and standard deviation are calculated. This is done, for example, in Fig. 5 of the main text as g_LR would overestimate the size of Δ_i due to asymmetries in the signal strength. In most of the data however, both nonlocal signals look similar and result in similar estimations for Δ_i even if the visibility of one of the signals is weak.

The gap in the superconducting shell Δ_SC is determined from the measurement in a similar way. Four traces at positive and negative bias are obtained from g_RL and g_LR, as shown in Fig. 3a. The top panel in Fig. 3b shows the traces as a function of super gate voltage V_SG. We determine Δ_SC by calculating their mean, which is shown in the bottom panel of Fig. 3b as the dark red curve. Similarly, the standard deviation is shown as the red shaded area.

* g.p.mazur@tudelft.nl

† l.p.kouwenhoven@tudelft.nl

‡ These authors contributed equally to this work

We note that the algorithm we apply to estimate Δ_{i} and Δ_{SC} does have its limitations. For instance, Δ_{SC} should remain constant but in some cases the nonlocal signal close to this value is weak in comparison to the rest of the trace. This usually happens in the weak-coupling regime, where the majority of the quasiparticle transport across the hybrid is carried by low-energy states. As a result, the outer edge of the nonlocal signal can become almost invisible. For example, this happens in the main text in Fig. 2 for the $8\,\mu$m long hybrid. We suspect that energy relaxation in the hybrid plays a role in this [1].

B. Extraction of the nonlocal slope

Estimation of Δ_{i} is challenging when the magnitude of the nonlocal signal is small, which is typically the case for long hybrids. In particular, problems arise when the induced gap is closed. This results in residual fluctuations as shown in Fig. 1 above $V_{\mathrm{SG}} > 6\,\mathrm{V}$. Thus, we always complement the gap estimation algorithm by looking at the nonlocal slopes S_{RL} and S_{LR}. In Fig. 4 we show an example of how these are obtained from the nonlocal data. Examples are shown for two linecuts, presented in the middle column (yellow) and right column (orange) for a large gap and a closed gap, respectively. We first apply a Savitzky-Golay filter, as is shown in dark green in Fig. 4b and d. We subsequently calculate the derivative of the filtered data, of which we take the value at zero bias to be the nonlocal slope. In Fig. 4c and e, we show the filtered data together with the tangent at zero bias as a dashed orange line. We see that the nonlocal slope at zero bias is significantly larger when the gap is closed. As can be seen in the various plots of the nonlocal slope in the main text, S_{RL} and S_{LR} behave similar in the sense that once the induced gap closes, both of them start to deviate from zero.

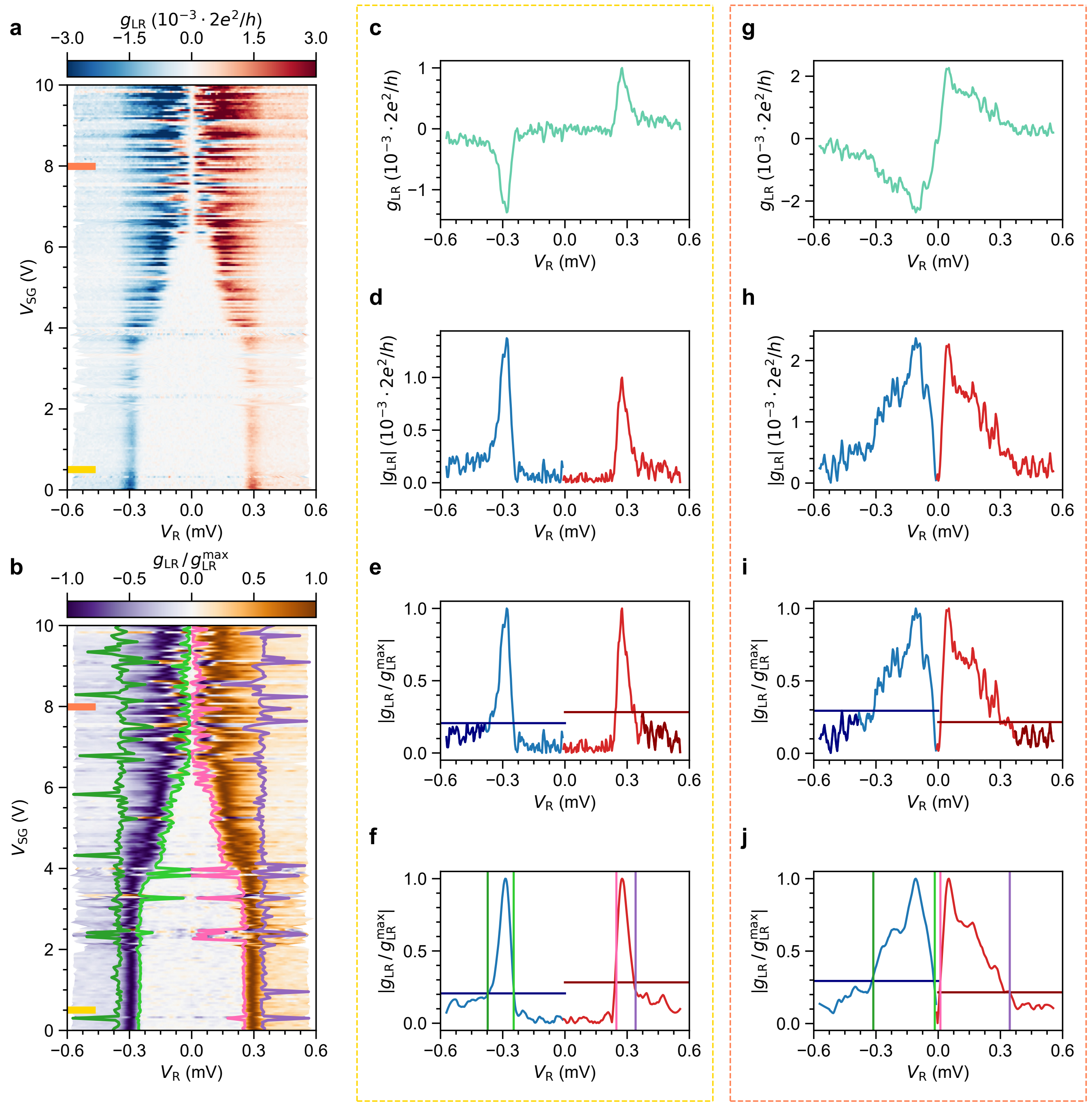

Supplementary Fig. 1. Example of the gap extraction algorithm used in this work, with data on device B (8 μm). Middle and right columns show linecuts taken at locations specified by the color bars in panel **a** for various stages during the analysis. **a** Nonlocal conductance matrix element g_{LR}. **b** g_{LR} after processing, including the estimates for Δ_i and Δ_{SC}. **c,g** Linecuts of g_{LR} taken with a large induced gap **c** and a closed gap **g**. **d,h** The g_{LR} data is split into positive (red) and negative (blue) bias values and the absolute value is taken. **e,i** The positive and negative bias traces are normalized by their maximum value. The out-of-gap signal is used to set separate thresholds for positive (dark red) and negative (dark blue) biases relative to the maximum of the signal. **f,j** The data is smoothened using a Savitzky-Golay filter. The thresholds obtained in panels **e,i** are used to determine the Δ_i for positive (pink) and negative (light green) biases, as well as for Δ_{SC} for positive (purple) and negative (dark green) biases.

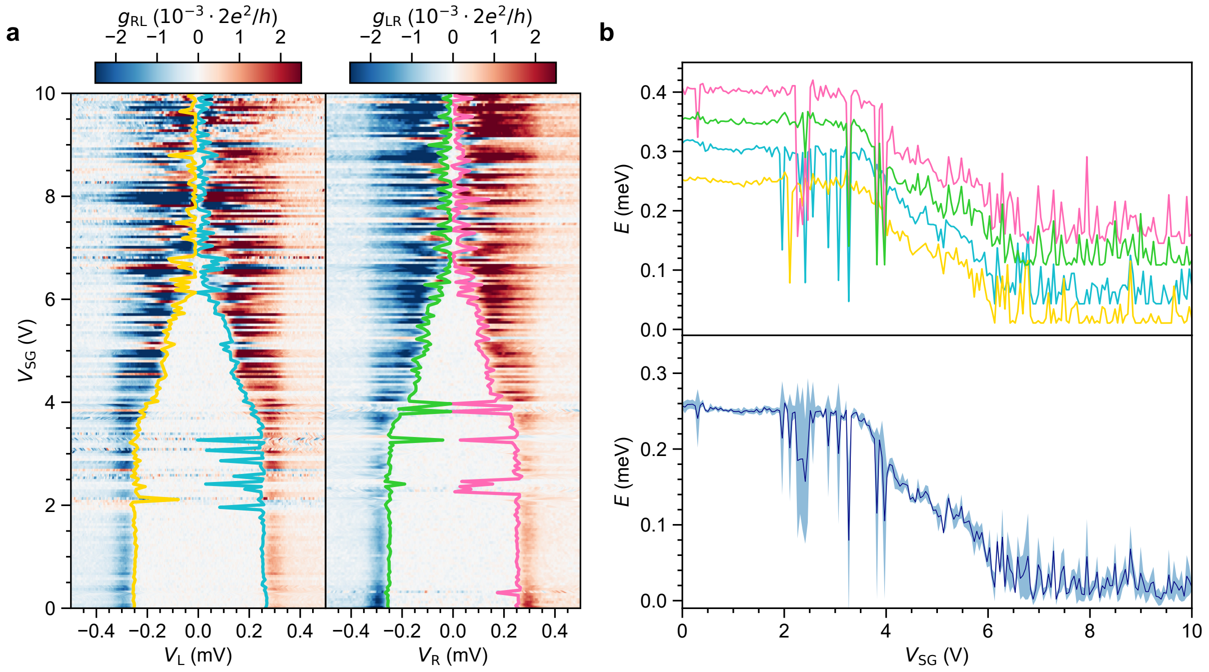

Supplementary Fig. 2. Example of the induced gap extraction. **a** $g_{\rm RL}$ and $g_{\rm LR}$ taken on device B, together with the four estimates for $\Delta_{\rm i}$: negative bias for $g_{\rm RL}$ (yellow) and $g_{\rm LR}$ (light green), and positive bias for $g_{\rm RL}$ (cyan) and $g_{\rm LR}$ (pink). **b** Top: the four estimated values of $\Delta_{\rm i}$, offset by 0.05 meV. Bottom: Mean of the four traces (dark blue) and their standard deviation (blue shaded area).

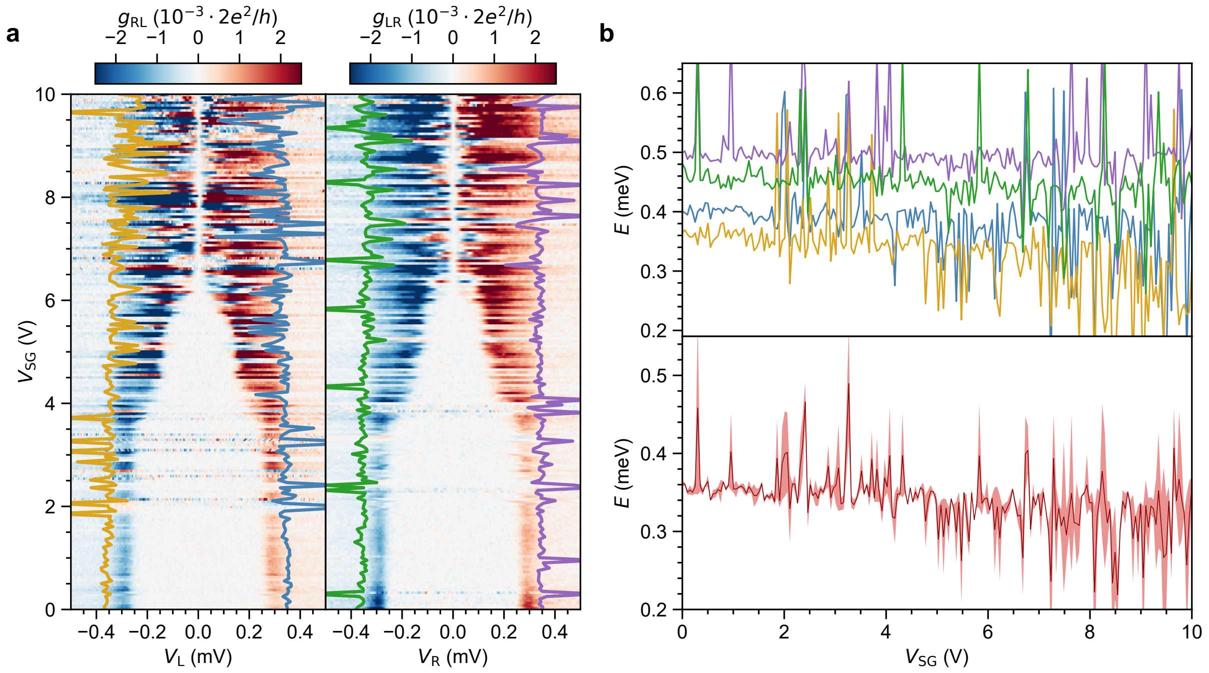

Supplementary Fig. 3. Example of extraction of the superconducting gap in the shell. **a** $g_{\rm RL}$ and $g_{\rm LR}$ taken on device B, together with the four estimates for $\Delta_{\rm SC}$: negative bias for $g_{\rm RL}$ (dark yellow) and $g_{\rm LR}$ (dark green), and positive bias for $g_{\rm RL}$ (blue) and $g_{\rm LR}$ (purple). **b** Top: the four estimated values of $\Delta_{\rm SC}$, offset by 0.05 meV. Bottom: Mean of the four traces (dark red) and their standard deviation (red shaded area).

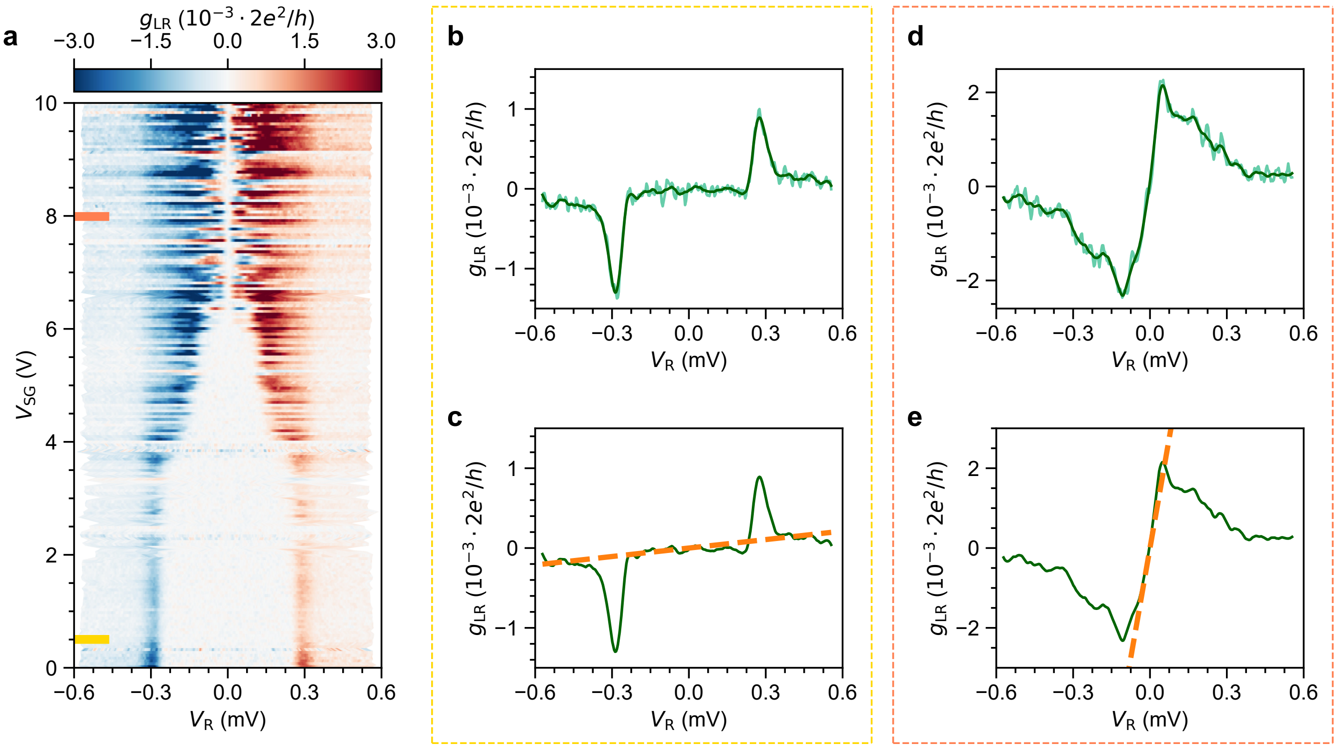

Supplementary Fig. 4. Example of the determination of the nonlocal slope. Middle and right columns show linecuts taken at locations specified by the color bars in panel **a** for various stages during the analysis. Linecuts are the same as the ones used in Fig. 1. **a** Nonlocal conductance matrix element g_{LR} taken on device B. **b,d** Linecuts of g_{LR} taken with a large induced gap **b** and a closed gap **d**. The signals are smoothened using a Savitzky-Golay filter, shown in dark green. **c,e** Filtered signals together with the tangent of the nonlocal slope at zero bias (orange dashed line).

II. ADDITIONAL DATA AND DISCUSSION

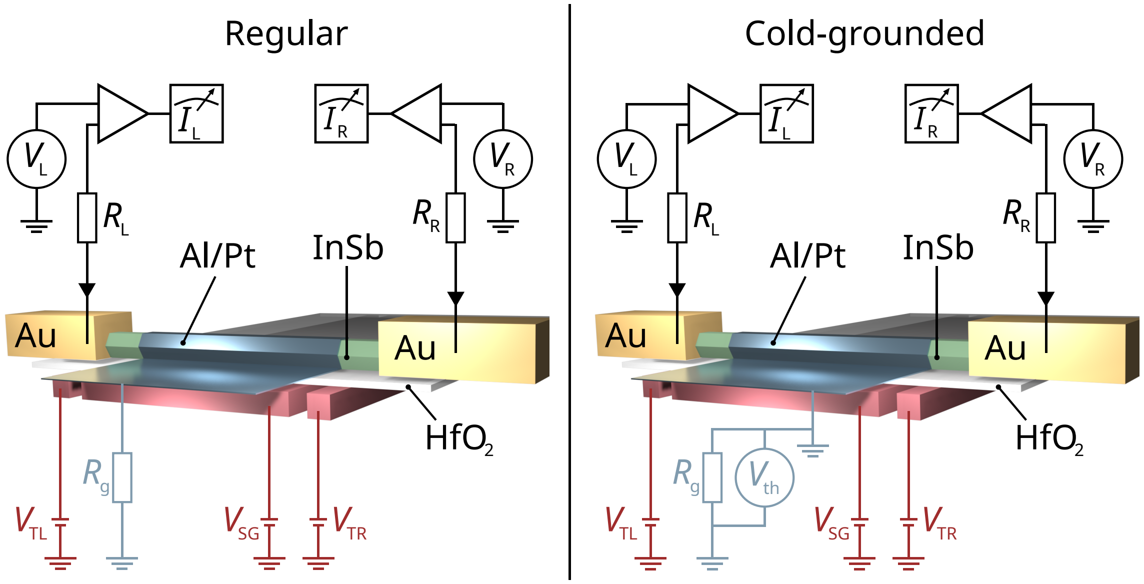

Supplementary Fig. 5. Schematic of the used measurement circuit, including fridge line resistance R_L,R_R and R_g. Left side shows the regular circuit used for device C, which has the fridge line resistance between the Al/Pt film and the ground. This results in potential voltage-divider effects, which need to be corrected. Right side shows the cold-grounded adaptation of the measurement circuit used for devices A and B. The cold grounding helps circumvent voltage-divider effects, but introduces a thermal voltage V_{th} on the applied biases. This is measured by also grounding the sample at room temperature and is actively compensated for during the measurements.

A. Overview of devices

In this work, we show data on six nanowire hybrids (see Fig.6). Two of these devices are presented in the main text, with additional data on those and three other devices in this section. In total, we fabricated 7 chips on which a total of 36 nanowires were cooled down for measurement. Due to fabrication issues, some of these chips were non-functional which resulted in 15 nanowires working (defined as fully gate-controllable and with a hard induced gap). We performed a detailed study on 11 of these nanowires, with hybrid lengths varying from 240 nm up to 8 μm. In this section, we present the full conductance matrices corresponding to the main text figures in Fig.11 and Fig.12. We also show two additional induced gap diagrams without a reopening of the induced gap in Fig.8, Fig.15. We elaborate on the soft gap versus hard gap nomenclature in Fig.9. We also show a comparison on the local spectra g_{LL} and g_{RR} for short and long wires in Fig.10. Finally, we give a representative device overview of hybrids for which we did not map out the induced gap diagram in Fig.7, Fig.13 and Fig.14.

We systematically observe the gate-tunable reduction of the induced gap in the explored nanowires. While in short devices Δ_i cannot be closed at zero field, we do observe that discrete states can cross zero energy. This is shown in Fig.16 and Fig.14**a-d**. In such devices, the induced gap consists of a collection of discrete Andreev bound states (ABS). We suspect that some of these states can acquire a finite charging energy as their wave function is pulled away from the semiconductor-superconductor interface due to strongly positive super gate voltages. The presence of a charging energy can allow them to cross zero energy [2], resembling the closing of the induced gap. However, this only happens for a finite range of gate voltages, in contrast to the long hybrids where the induced gap can be fully closed. It is not yet clear why this happens for some short devices (device E and F) but not for others (device A and D).

Similarly, we observe a strong reduction of the induced critical field as a function of super gate voltage. However, in some devices the critical induced field in the strong-coupling regime can be significantly lower than the critical field of the superconducting shell. For example, Fig.7**e-h** shows that the induced critical field in the strong-coupling regime for device A reaches only $B_{\parallel}^{c} = 1.5\,$T, while Δ_{SC} closes only above $B > 4\,$T.

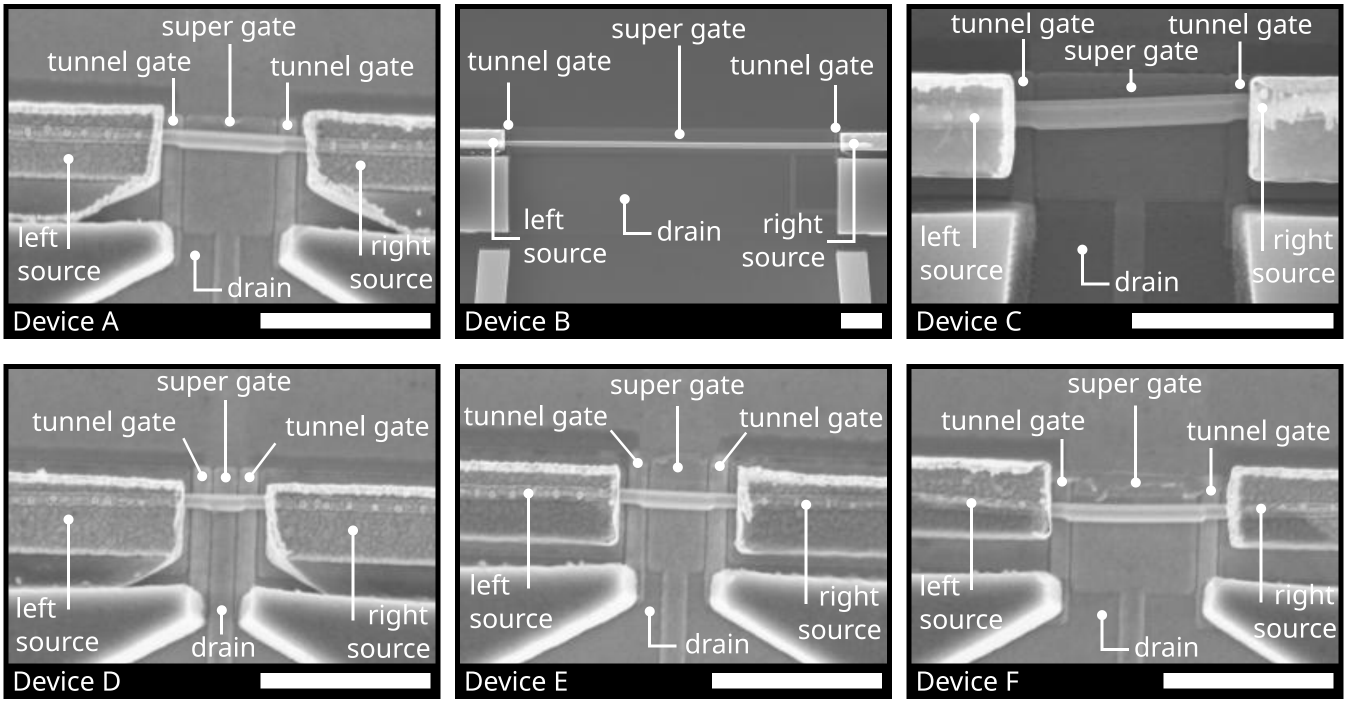

Supplementary Fig. 6. SEM images of the devices presented in this work. The white scalebar in each panel represents 1 μm. The top row shows devices B and C which are used for the main text. The length of the hybrid section of each device is: Device A - length: 640 nm, diameter: 79 nm, Device B - length: 8 μm, diameter: 120 nm, Device C - length: 1 μm, diameter: 126 nm, Device D - length: 240 nm, diameter: 75 nm, Device E - length: 450 nm, diameter: 77 nm, Device F - length: 840 nm, diameter: 81 nm.

B. Hard and soft induced superconducting gaps

In literature on the proximity effect, various definitions are used to judge on the presence of an induced gap in a proximitized system. An induced gap is typically referred to as a hard gap, while the absence of a gap (but with a reduced conductance at low bias) is referred to as a soft gap. Typically, a local signal is used and the sub-gap to out-of-gap ratio is used to claim a hard induced gap. However, such a metric is unable to distinguish between the presence of a true induced gap or, for example, the reduction of local sub-gap conductance due to coulomb blockade in the semiconducting junctions. Fortunately, nonlocal conductance can be used to determine if an induced gap is present in a hybrid. For example, in Fig. 9 we show additional data on device B where a voltage on the super gate is used to close the induced gap. In Fig. 9e, we show the conductance matrix at $V_{\mathrm{SG}} = 6.05\,\mathrm{V}$. Here, Δ_{i} is on the order of $\Delta_{\mathrm{i}} = 120\,\mu\mathrm{eV}$, which is visible in the nonlocal conductances g_{RL} and g_{LR} as the flat part between the two peaks. At the same time, the local conductances g_{LL} and g_{RR} show a broadening of the coherence peaks which corresponds to the reduction of Δ_{i}. At higher super gate voltages, Δ_{i} closes. For example, Fig. 9f shows the conductance matrix at $V_{\mathrm{SG}} = 7.45\,\mathrm{V}$. From the local signal g_{LL} it may appear as if the hybrid still has a hard gap, while g_{RR} shows a finite sub-gap conductance. In this case, the nonlocal signals g_{RL} and g_{LR} both show an absence of a flat part between the two peaks (i.e. a finite nonlocal slope at zero bias) which confirms that the hybrid is gapless.

We note that the induced gap in short hybrids does not close at zero magnetic field, as can be seen for example in Fig. 7. This is a consequence of the short length of this hybrid: while states without any semiconductor-superconductor coupling may form in the weak-coupling regime, in practice these can obtain a finite energy gap if they are allowed to mix with proximitized states. Such mixing can occur due to disorder and, in this particular case of short hybrids, due to the presence of tunnel junctions at the ends of the hybrid segment.

C. Absence of end-to-end correlation in local spectra

One striking feature we observe is that the local conductance spectra g_{LL} and g_{RR} do not seem to change significantly as the length of a nanowire hybrid is increased. In Fig.10, we compare the spectra of device F (800 nm) and device B (8 μm) in the weak-coupling regime. The local spectra of device F (Fig.10**a,b**) show the field evolution of a discrete number of states, as expected for a short nanowire hybrid [3]. For device B, the increased length is expected to reduce the level spacing of these states such that they form a continuum. However, the local spectra show a similar field evolution of a discrete number of states (Fig.10**g,h**). In addition, the local spectra g_{LL} and g_{RR} generically appear to be uncorrelated. These observations both indicate that the tunnel junctions significantly disrupt the potential profile at the ends of the hybrid, leading to the formation of localized states. This suggests that the local spectra are only looking at an effective short nanowire segment located in the vicinity of the tunnel junctions. Such a scenario could potentially give rise to dark Majoranas, which would not show up as zero-bias peaks in the local end spectra but still be visible in the nonlocal signals as a reopening in the bulk of the nanowire. This in contrast to the formation of quasi-Majoranas, which form on the smooth potential of the tunnel barriers - resulting in a zero-bias peak without a reopening of the bulk gap.

D. Interpretation of nonlocal transport measurements

In this work, we use nonlocal spectroscopy to investigate the bulk properties of three-terminal InSb/Al/Pt nanowire hybrids. In particular, nonlocal transport is facilitated through the density of states between Δ_{SC} and Δ_{i}. However, several processes complicate this simple picture. For example, the visibility of nonlocal signals is affected strongly by the non-ideal injection and detection processes in the tunnel junctions [1]. Moreover, relaxation from above to below Δ_{SC} is sometimes visible in the nonlocal spectra [1]. In addition, Δ_{i} can potentially vary along the length of the hybrid. As a result, the nonlocal signal may reflect the largest induced gap somewhere in the bulk. However, nonlocal transport is likely insensitive to fluctuations of Δ_{i} on a short length scale as quasiparticles can cross such areas through various tunneling mechanisms. Despite the above-mentioned complications, we systematically observe a good correspondence between the two nonlocal signals g_{RL} and g_{LR}, which supports the assumption that nonlocal transport can be used to evaluate bulk properties.

Device A

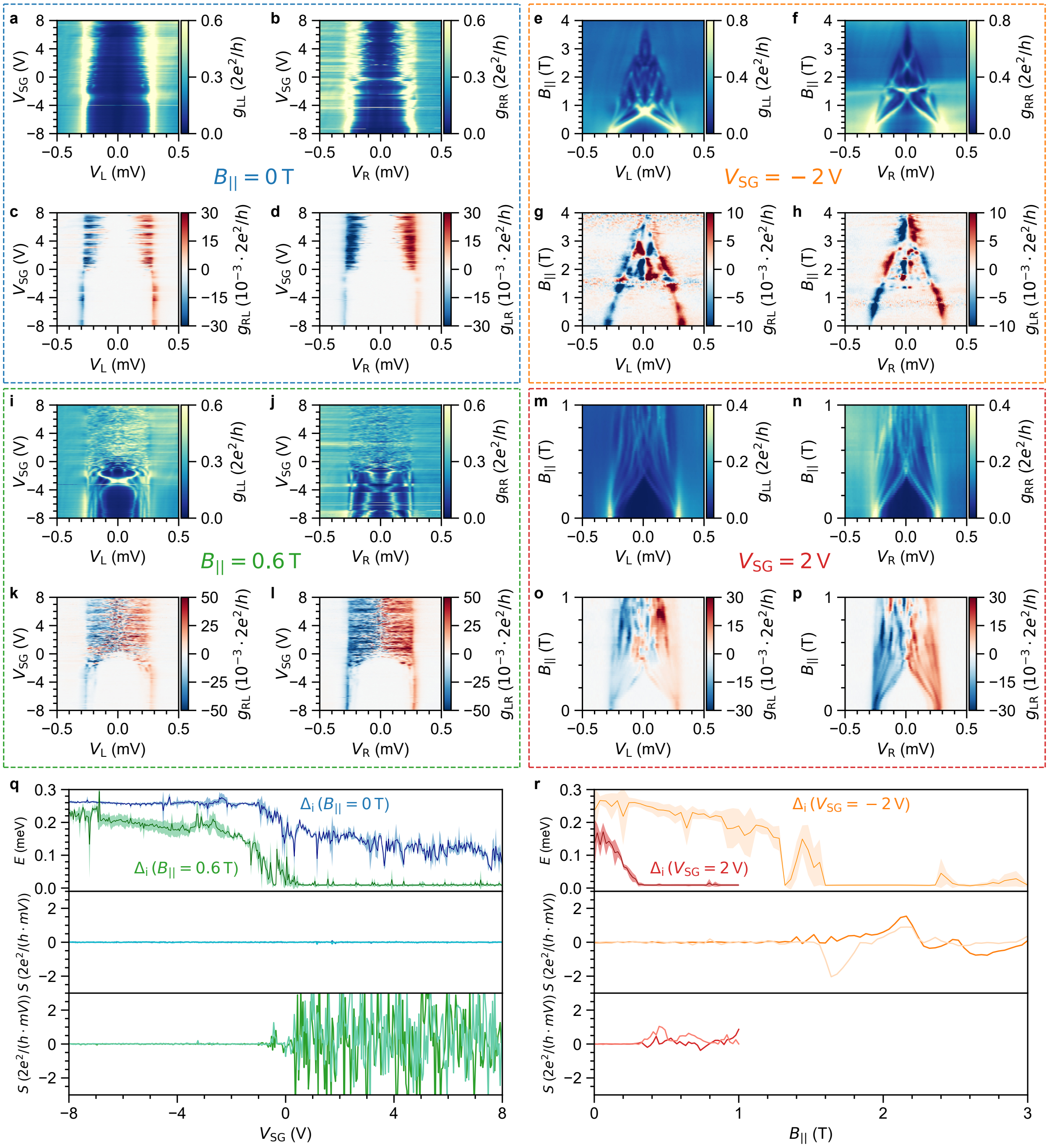

Supplementary Fig. 7. Representative overview of device A (640 nm). **a-d** (Blue) Conductance matrix as a function of V_{SG} taken at $B_{\parallel} = 0\,\mathrm{T}$. **e-h** (Orange) Conductance matrix as a function of $B_{\parallel}$ in the strong-coupling regime, taken at $V_{SG} = -2\,\mathrm{V}$. **i-l** (Green) Conductance matrix as a function of V_{SG} taken at $B_{\parallel} = 0.6\,\mathrm{T}$. **m-p** (Red) Conductance matrix as a function of $B_{\parallel}$ in the weak-coupling regime, taken at $V_{SG} = 2\,\mathrm{V}$. **q** Top: Δ_i as function of V_{SG} at $B_{\parallel} = 0\,\mathrm{T}$ (blue) and $B_{\parallel} = 0.6\,\mathrm{T}$ (green). Middle: Nonlocal slope as function of V_{SG} at $B_{\parallel} = 0\,\mathrm{T}$, indicating the nanowire maintains an induced gap at all gate values. Bottom: Nonlocal slope as function of V_{SG} at $B_{\parallel} = 0.6\,\mathrm{T}$, showing the induced gap closing around $V_{SG} = -1\,\mathrm{V}$. **r** Top: Δ_i as function of $B_{\parallel}$ at $V_{SG} = -2\,\mathrm{V}$ (orange) and $V_{SG} = +2\,\mathrm{V}$ (red). Middle: Nonlocal slope as function of $B_{\parallel}$ taken at $V_{SG} = -2\,\mathrm{V}$, showing the induced gap closing at $B_{\parallel}^{c} = 1.5\,\mathrm{T}$. Bottom: Nonlocal slope as function of $B_{\parallel}$ taken at $V_{SG} = 2\,\mathrm{V}$, showing the induced gap closing at $B_{\parallel}^{c} = 0.3\,\mathrm{T}$.

Device B

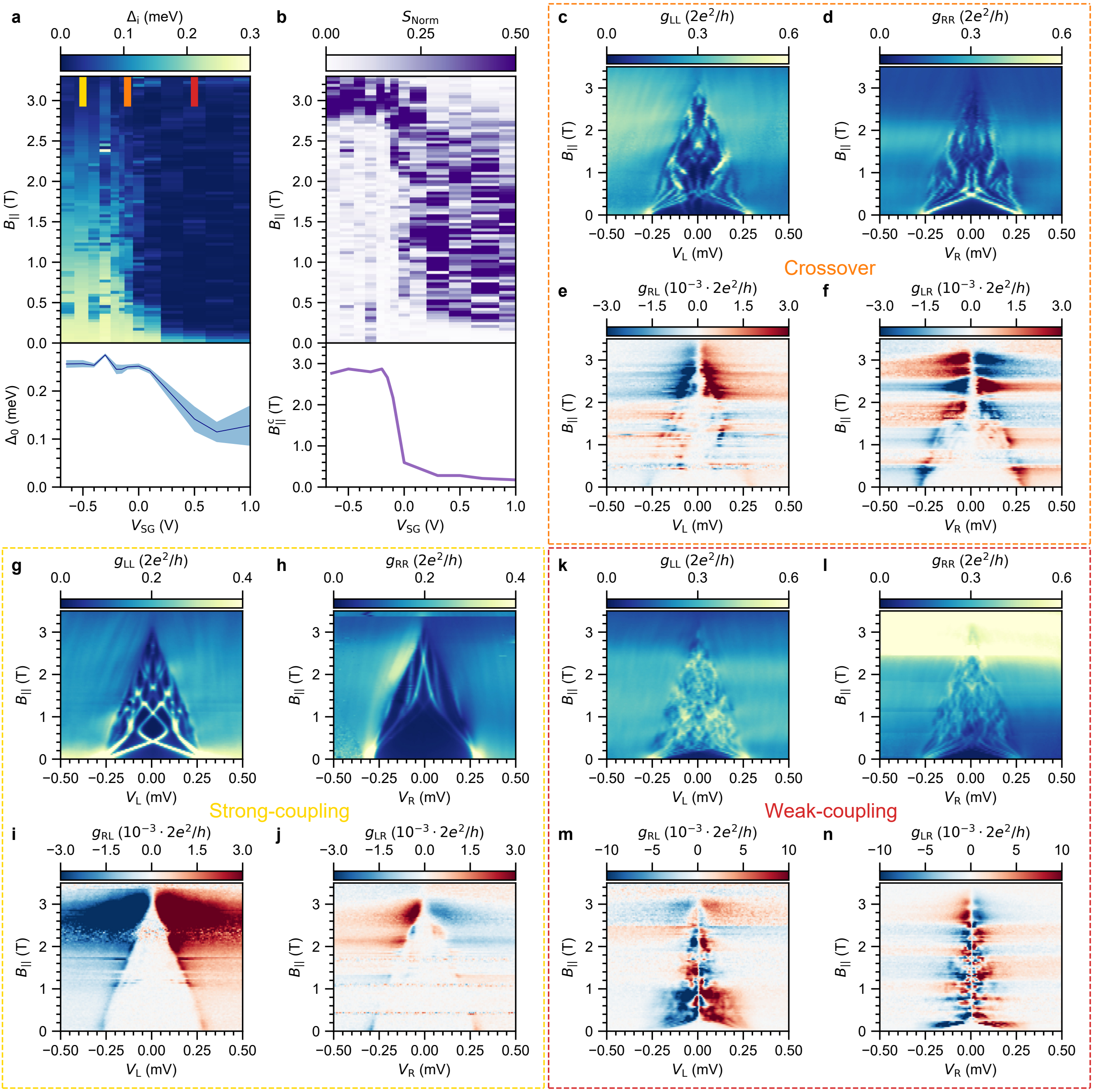

Supplementary Fig. 8. Induced gap and nonlocal slope diagrams for device B (8 μm long hybrid). **a** Top: Δ_i as a function of V_{SG} and $B_{\parallel}$. Bottom: Δ_i at $B_{\parallel} = 0$ T. We note that the gate voltage for which Δ_i starts to decrease is quite different from the data presented in the main text (Fig. 2**c-d**). This is the result of dielectric hysteresis, as the gate voltages are swept over a large voltage range. **b** Top: S_{Norm} as a function of V_{SG} and $B_{\parallel}$. Bottom: $B_{\parallel}^c$ as a function of V_{SG}. **c-f** (Orange) Conductance matrix as a function of $B_{\parallel}$ taken at $V_{SG} = -0.1$ V. **g-j** (Yellow) Conductance matrix as a function of $B_{\parallel}$ taken at $V_{SG} = -0.5$ V. **k-n** (Red) Conductance matrix as a function of $B_{\parallel}$ taken at $V_{SG} = 0.5$ V.

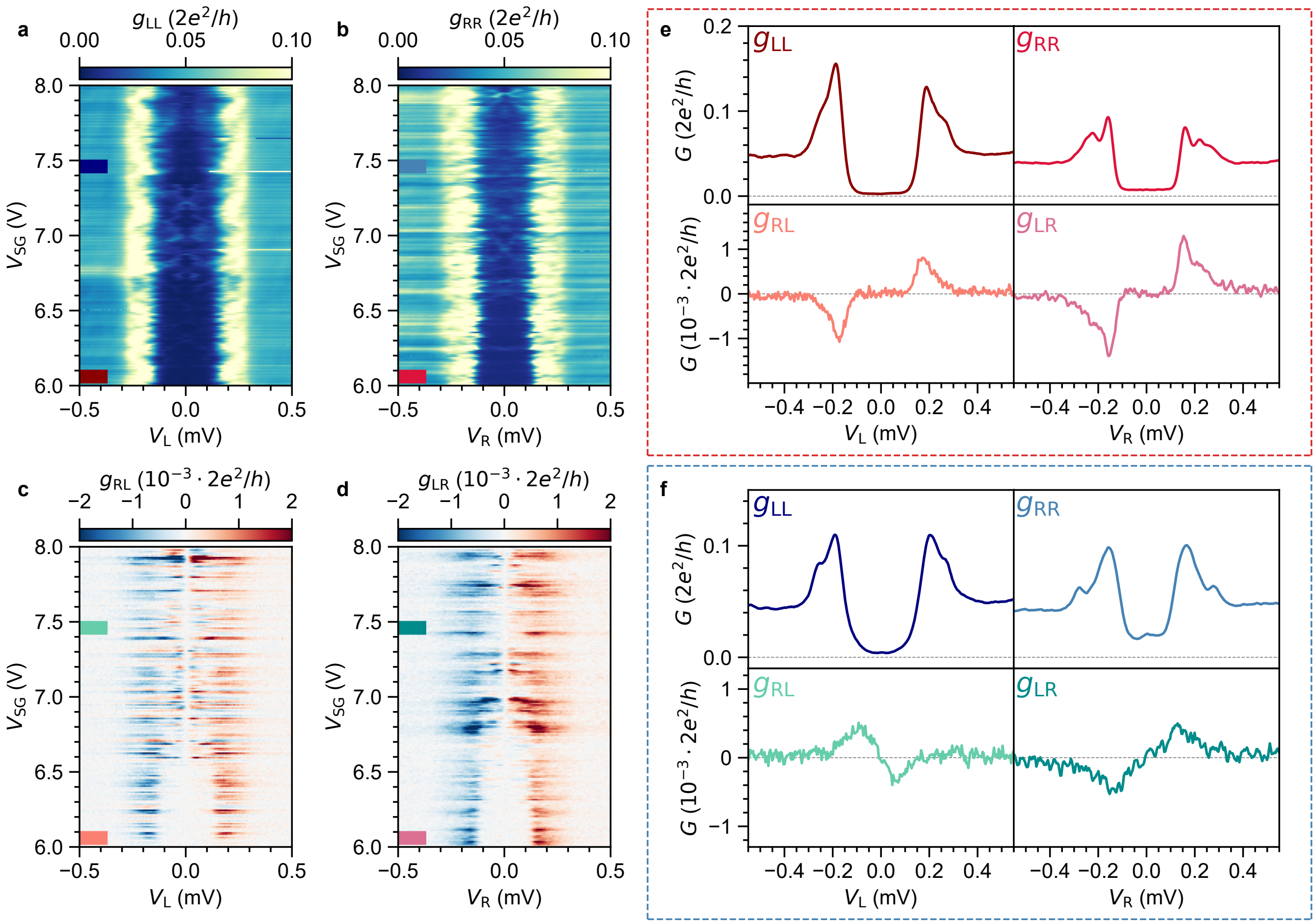

Supplementary Fig. 9. Full conductance matrix of device B ($8\,\mu$m) as a function of V_{SG} taken at $B_\parallel = 0\,$T. **a,b** Local conductances g_{LL} and g_{RR}. **c,d** Nonlocal conductances g_{RL} and g_{LR}. **e** Linecuts of the local and nonlocal spectra taken at $V_{SG} = 6.05\,$V where the nanowire has a (hard) induced gap. **f** Linecuts of the local and nonlocal spectra taken at $V_{SG} = 7.45\,$V where the induced gap in the nanowire is closed (i.e. a soft gap).

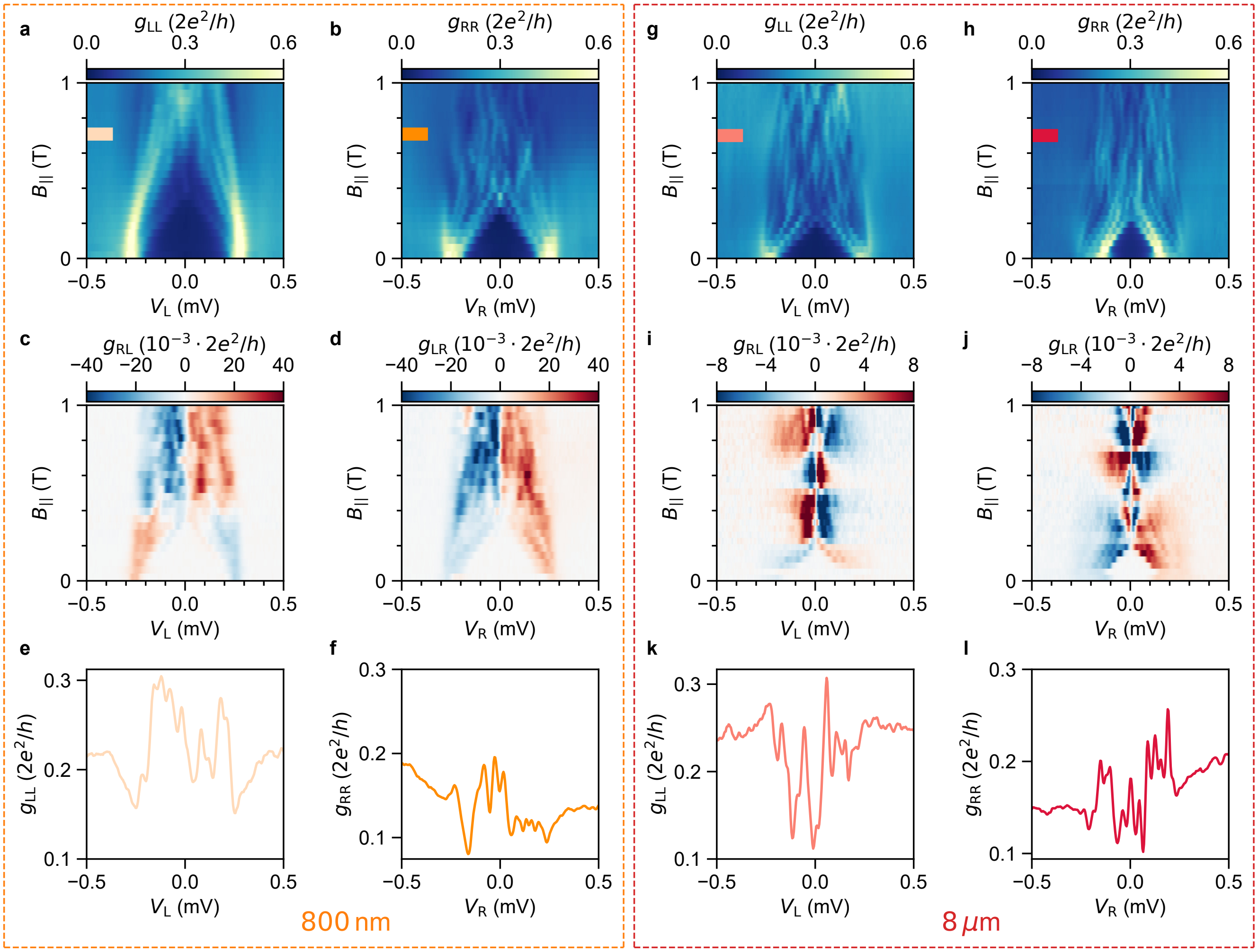

Supplementary Fig. 10. Comparison of the spectra between short (device F, 800 nm) and long (device B, 8 μm) hybrids in the weak-coupling regime. Both short (left) and long (right) nanowires form similar-sized fragmented segments in the nanowire near the junctions, as a result of the identical feature size of the tunnel gate geometry. Accordingly, both spectra exhibit a similar number of discrete states which are formed near the junctions. **a-d** Conductance matrix of device F as a function of $B_{\|}$ in the weak-coupling regime, taken at $V_{\mathrm{SG}} = 1.75\,\mathrm{V}$. **e,f** linecuts of the local spectra g_{LL} and g_{RR} taken at $B_{\|} = 0.7\,\mathrm{T}$. **g-j** Conductance matrix of device B as a function of $B_{\|}$ in the weak-coupling regime, taken at $V_{\mathrm{SG}} = 1\,\mathrm{V}$. **k,l** linecuts of the local spectra g_{LL} and g_{RR} taken at $B_{\|} = 0.7\,\mathrm{T}$.

Device C

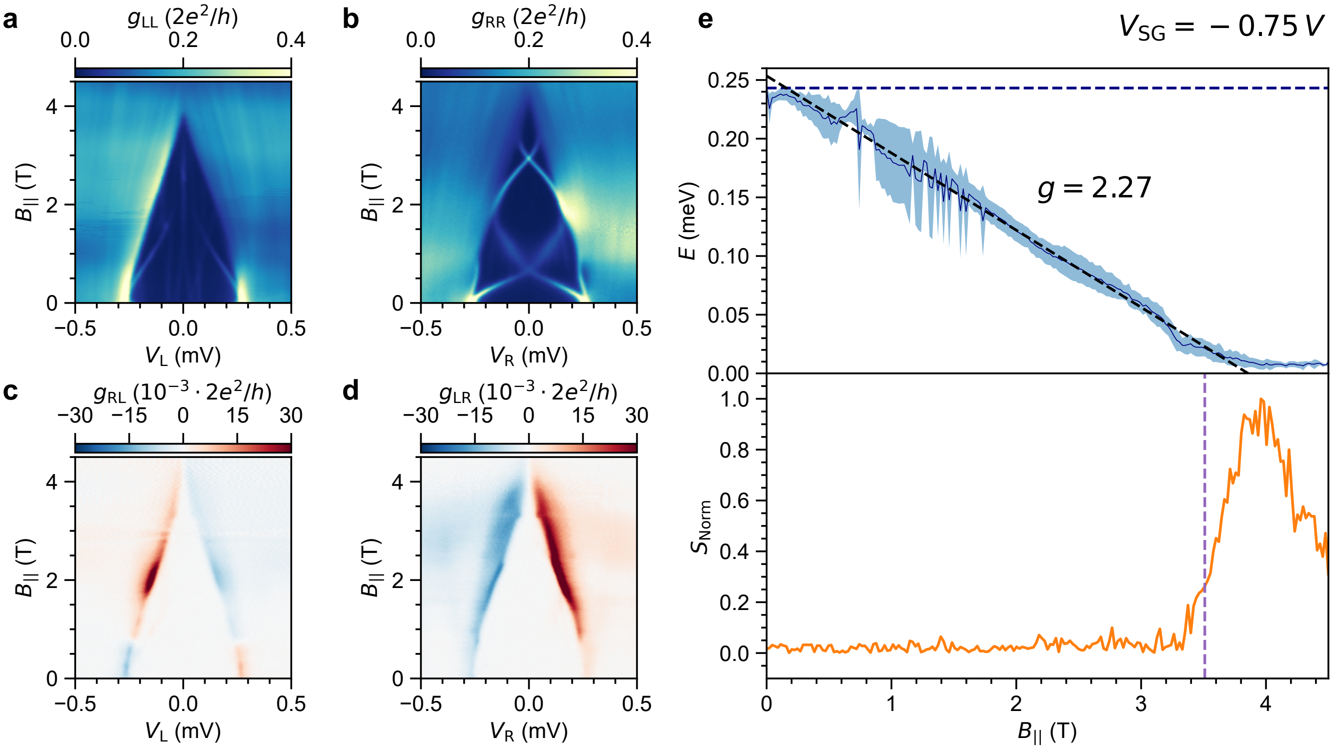

Supplementary Fig. 11. Full conductance matrix of device C ($1\,\mu$m) as a function of parallel magnetic field in the strong-coupling regime, corresponding to panels **a,b** of Fig. 3 in the main text. **a,b** Local conductances $g_{\rm LL}$ and $g_{\rm RR}$. **c,d** Nonlocal conductances $g_{\rm RL}$ and $g_{\rm LR}$. **e** Top: $\Delta_{\rm i}$ as a function of parallel magnetic field. The dashed black line indicates the fit of the Zeeman energy to the linear part of the data with $g = 2.27$. The dashed blue line indicates the zero-field induced gap Δ_0. Bottom: Normalized nonlocal slope $S_{\rm Norm} = |S_{\rm RL} S_{\rm LR}|/\sqrt{|S_{\rm RL} S_{\rm LR}|}$ normalized by its maximum value. The dashed purple line indicates the estimated induced critical field $B_{\|}^{\rm c}$.

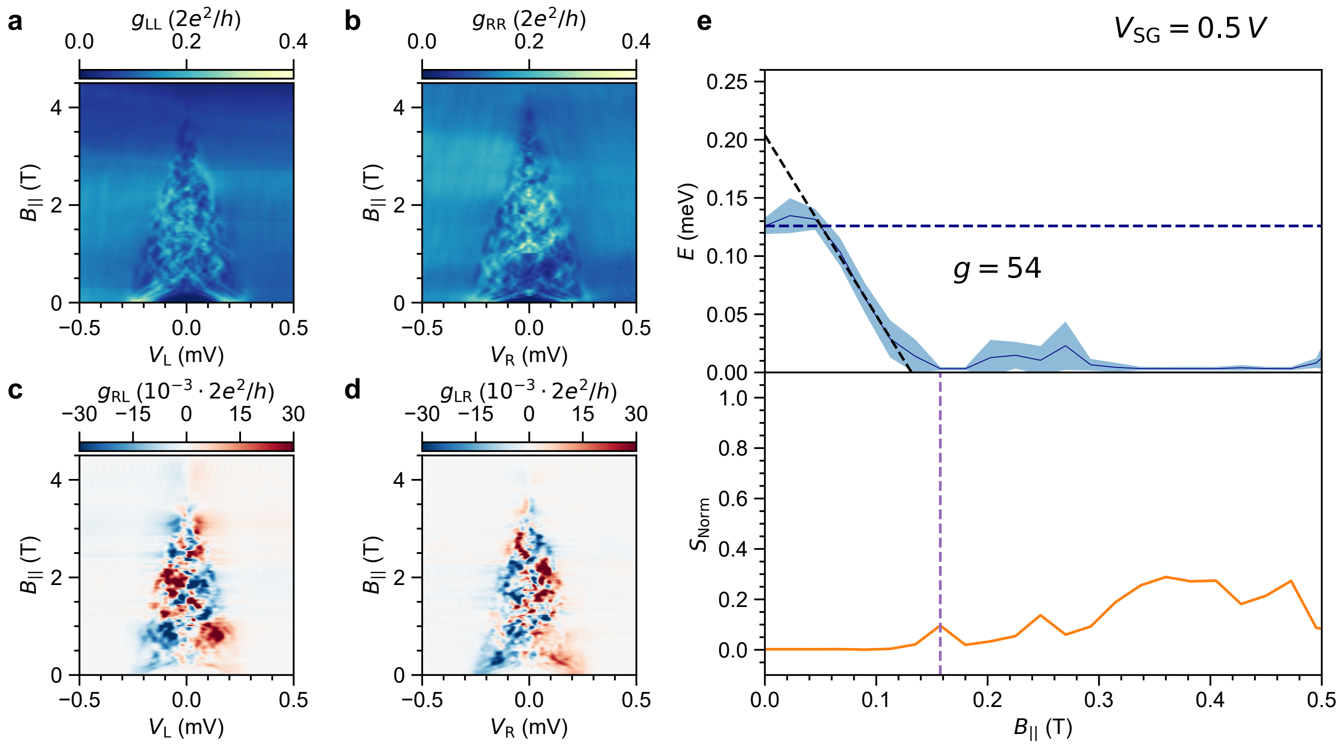

Supplementary Fig. 12. Full conductance matrix of device C ($1\,\mu$m) as a function of parallel magnetic field in the weak-coupling regime, corresponding to panels **c,d** of Fig. 3 in the main text. **a,b** Local conductances g_{LL} and g_{RR}. **c,d** Nonlocal conductances g_{RL} and g_{LR}. **e** Top: Δ_{i} as a function of parallel magnetic field. The dashed black line indicates the fit of the Zeeman energy to the linear part of the data with $g = 54$. The dashed blue line indicates the zero-field induced gap Δ_0. Bottom: Normalized nonlocal slope $S_{\mathrm{Norm}} = |S_{\mathrm{RL}}S_{\mathrm{LR}}|/\sqrt{|S_{\mathrm{RL}}S_{\mathrm{LR}}|}$ normalized by its maximum value. The dashed purple line indicates the estimated induced critical field $B_{\|}^{\mathrm{c}}$

Device D

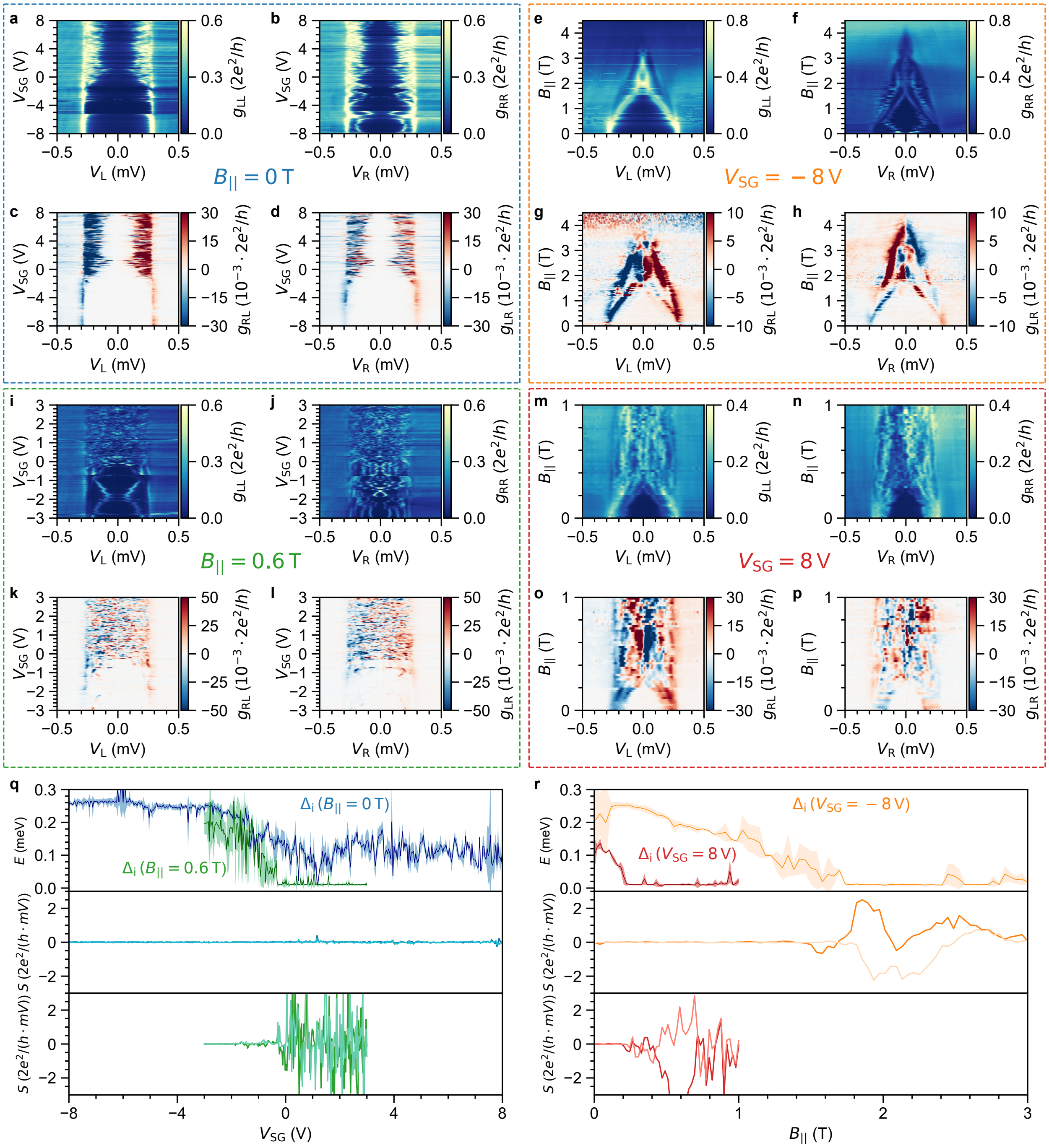

Supplementary Fig. 13. Representative overview of device D (240 nm hybrid). **a-d** (Blue) Conductance matrix as a function of V_{SG} taken at $B_{\|} = 0\,\mathrm{T}$. **e-h** (Orange) Conductance matrix as a function of $B_{\|}$ in the strong-coupling regime, taken at $V_{\mathrm{SG}} = -8\,\mathrm{V}$. **i-l** (Green) Conductance matrix as a function of V_{SG} taken at $B_{\|} = 0.6\,\mathrm{T}$. **m-p** (Red) Conductance matrix as a function of $B_{\|}$ in the weak-coupling regime, taken at $V_{\mathrm{SG}} = 8\,\mathrm{V}$. **q** Top: Δ_{i} as function of V_{SG} at $B_{\|} = 0\,\mathrm{T}$ (blue) and $B_{\|} = 0.6\,\mathrm{T}$ (green). Middle: Nonlocal slope as function of V_{SG} at $B_{\|} = 0\,\mathrm{T}$, indicating the nanowire maintains an induced gap at all gate values. Bottom: Nonlocal slope as function of V_{SG} at $B_{\|} = 0.6\,\mathrm{T}$, showing the induced gap closing around $V_{\mathrm{SG}} = -0.5\,\mathrm{V}$. **r** Top: Δ_{i} as function of $B_{\|}$ at $V_{\mathrm{SG}} = -8\,\mathrm{V}$ (orange) and $V_{\mathrm{SG}} = -8\,\mathrm{V}$ (red). Middle: Nonlocal slope as function of $B_{\|}$ taken at $V_{\mathrm{SG}} = -8\,\mathrm{V}$, showing the induced gap closing at $B_{\|}^{\mathrm{c}} = 1.6\,\mathrm{T}$. Bottom: Nonlocal slope as function of $B_{\|}$ taken at $V_{\mathrm{SG}} = 8\,\mathrm{V}$, showing the induced gap closing at $B_{\|}^{\mathrm{c}} = 0.2\,\mathrm{T}$.

Device E

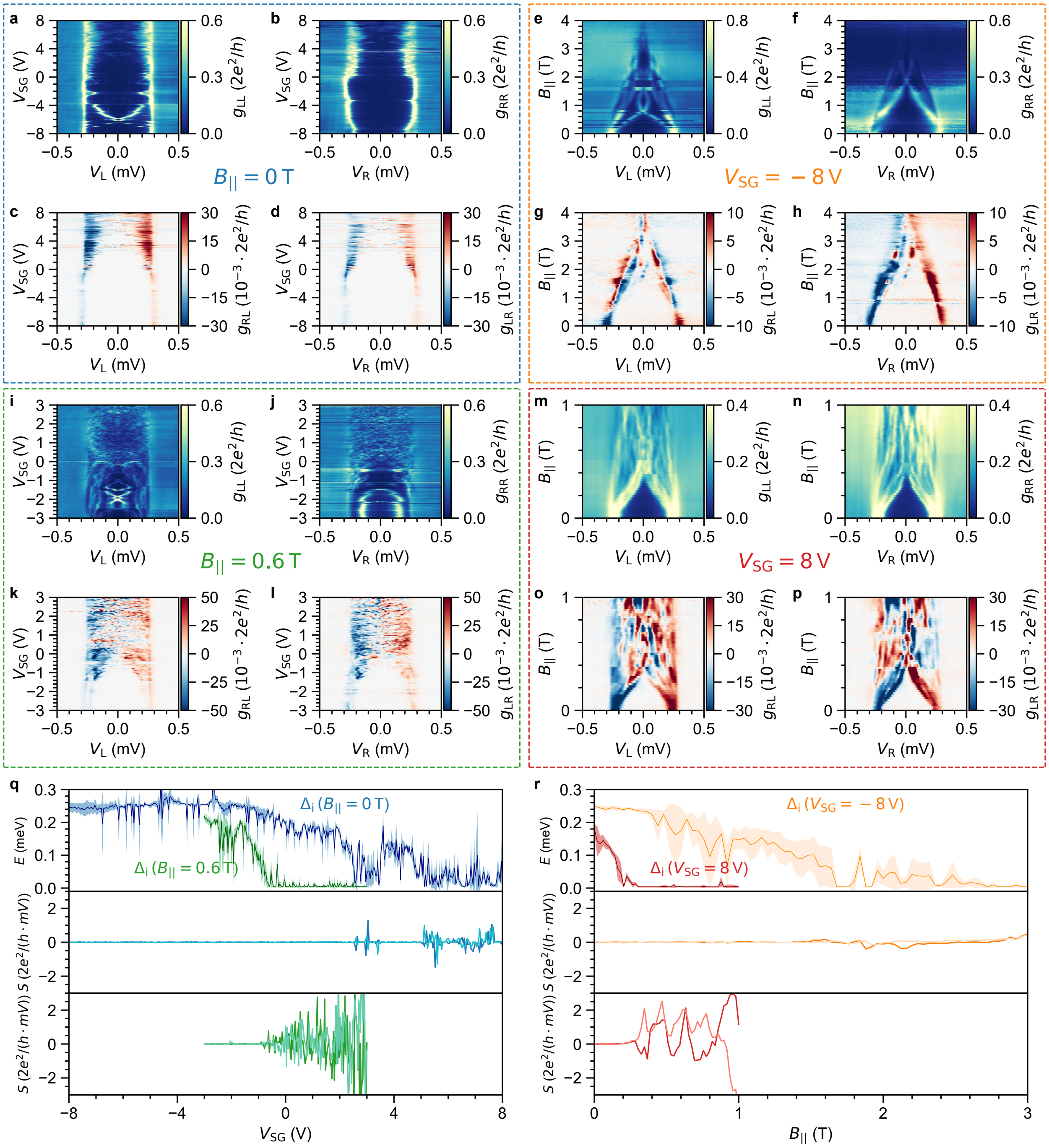

Supplementary Fig. 14. Representative overview of device E (450 nm hybrid). **a-d** (Blue) Conductance matrix as a function of V_{SG} taken at $B_{\|} = 0\,\mathrm{T}$. **e-h** (Orange) Conductance matrix as a function of $B_{\|}$ in the strong-coupling regime, taken at $V_{SG} = -8\,\mathrm{V}$. **i-l** (Green) Conductance matrix as a function of V_{SG} taken at $B_{\|} = 0.6\,\mathrm{T}$. **m-p** (Red) Conductance matrix as a function of $B_{\|}$ in the weak-coupling regime, taken at $V_{SG} = 8\,\mathrm{V}$. **q** Top: Δ_i as function of V_{SG} at $B_{\|} = 0\,\mathrm{T}$ (blue) and $B_{\|} = 0.6\,\mathrm{T}$ (green). Middle: Nonlocal slope as function of V_{SG} at $B_{\|} = 0\,\mathrm{T}$, showing an occational closing of the induced gap due to discrete states crossing zero energy. Bottom: Nonlocal slope as function of V_{SG} at $B_{\|} = 0.6\,\mathrm{T}$, showing the induced gap closing around $V_{SG} = -1\,\mathrm{V}$. **r** Top: Δ_i as function of $B_{\|}$ at $V_{SG} = -8\,\mathrm{V}$ (orange) and $V_{SG} = -8\,\mathrm{V}$ (red). Middle: Nonlocal slope as function of $B_{\|}$ taken at $V_{SG} = -8\,\mathrm{V}$, showing the induced gap closing at $B_{\|}^c = 1.6\,\mathrm{T}$. Bottom: Nonlocal slope as function of $B_{\|}$ taken at $V_{SG} = 8\,\mathrm{V}$, showing the induced gap closing at $B_{\|}^c = 0.23\,\mathrm{T}$.

Device F

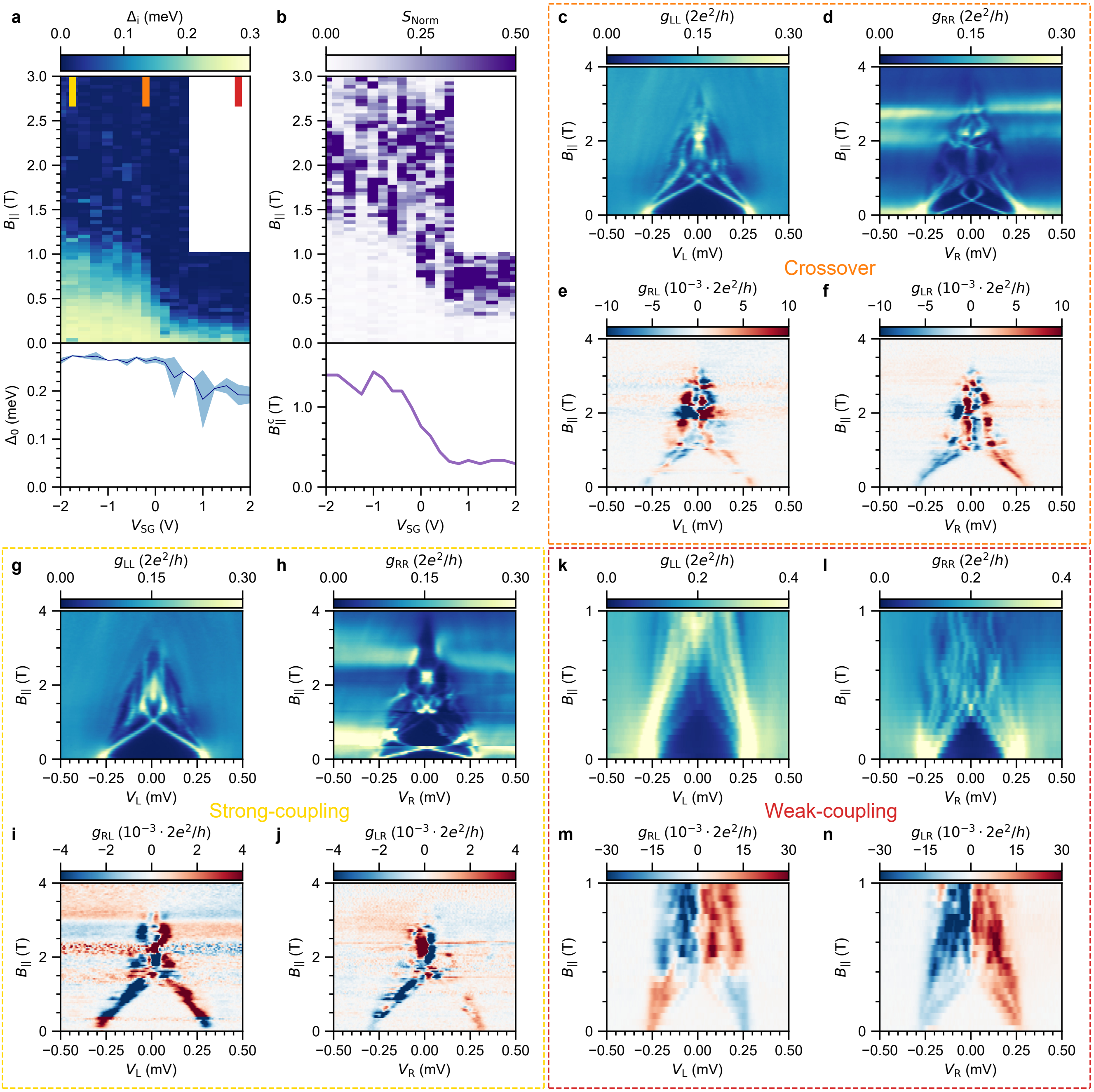

Supplementary Fig. 15. Induced gap and nonlocal slope diagrams for device F (800 nm long hybrid). **a** Top: Δ_i as a function of V_{SG} and $B_{\parallel}$. Bottom: Δ_i at $B_{\parallel} = 0$ T. **b** Top: Normalized nonlocal slope S_{Norm} as a function of V_{SG} and $B_{\parallel}$. Bottom: Parallel induced critical field $B^c_{\parallel}$ as a function of V_{SG}. **c-f** (Orange) Conductance matrix as a function of $B_{\parallel}$ taken at $V_{SG} = -0.1$ V. **g-j** (Yellow) Conductance matrix as a function of $B_{\parallel}$ taken at $V_{SG} = -1.75$ V. **k-n** (Red) Conductance matrix as a function of $B_{\parallel}$ taken at $V_{SG} = 1.75$ V.

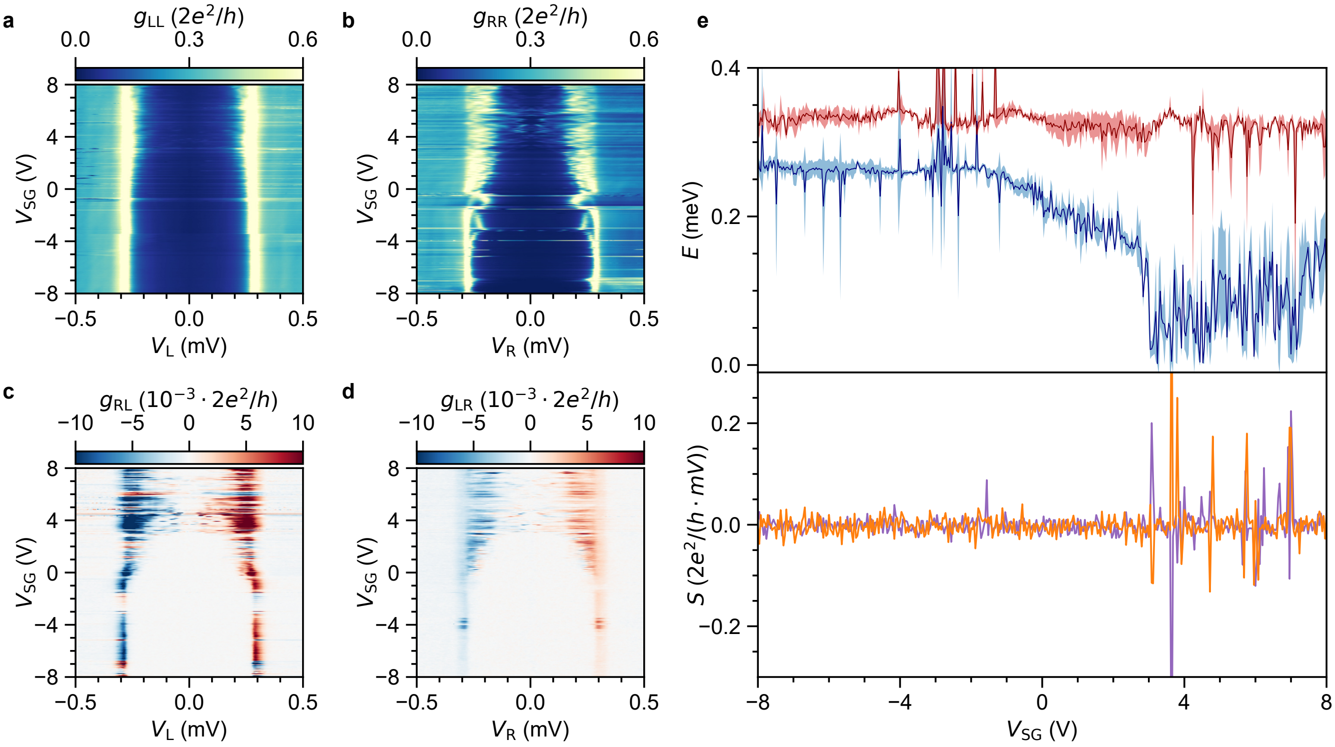

Supplementary Fig. 16. Full conductance matrix of device F as a function of V_{SG} taken at $B_{\parallel} = 0\,\mathrm{T}$. **a,b** Local conductances g_{LL} and g_{RR}. **c,d** Nonlocal conductances g_{RL} and g_{LR}. **e** Top: Δ_i (blue) and Δ_{SC} (red) as a function of V_{SG}. Bottom: calculated nonlocal slope at zero bias for g_{RL} (purple) and g_{LR} (orange). Here, Δ_i occasionally reaches zero but always returns to a finite value. We speculate this is the result of individual states in the hybrid attaining a finite charging energy due.

[1] G. Wang, T. Dvir, N. van Loo, G. P. Mazur, S. Gazibegovic, G. Badawy, E. P. A. M. Bakkers, L. P. Kouwenhoven, and G. de Lange, Nonlocal measurement of quasiparticle charge and energy relaxation in proximitized semiconductor nanowires using quantum dots, Phys. Rev. B **106**, 064503 (2022).

[2] J. Pillet, C. Quay, P. Morfin, C. Bena, A. L. Yeyati, and P. Joyez, Andreev bound states in supercurrent-carrying carbon nanotubes revealed, Nature Physics **6**, 965 (2010).

[3] T. D. Stanescu, R. M. Lutchyn, and S. D. Sarma, Dimensional crossover in spin-orbit-coupled semiconductor nanowires with induced superconducting pairing, Physical Review B **87**, 094518 (2013).
